# Lipid Coating Modulates Effects of Nanoceria on Oxidative Metabolism in Human Embryonic Lung Fibroblasts: A Case of Cardiolipin

**DOI:** 10.3390/biom15010053

**Published:** 2025-01-02

**Authors:** Elena V. Proskurnina, Madina M. Sozarukova, Elizaveta S. Ershova, Ekaterina A. Savinova, Larisa V. Kameneva, Natalia N. Veiko, Maria A. Teplonogova, Vladimir P. Saprykin, Vladimir K. Ivanov, Svetlana V. Kostyuk

**Affiliations:** 1 Research Centre for Medical Genetics, ul. Moskvorechye 1, Moscow 115522, Russia; es-ershova@rambler.ru (E.S.E.); savinova.ekaterina96@yandex.ru (E.A.S.); larisa.kameneva@yandex.ru (L.V.K.); satelit32006@yandex.ru (N.N.V.); svet-vk@yandex.ru (S.V.K.); 2 Kurnakov Institute of General and Inorganic Chemistry of the Russian Academy of Sciences, Leninskii Prospect 31, Moscow 119071, Russia; s_madinam@bk.ru (M.M.S.); ma_teplonogova@igic.ras.ru (M.A.T.); van@igic.ras.ru (V.K.I.); 3 Faculty of Biotechnology and Fisheries, K.G. Razumovsky Moscow State University of Technologies and Management, Zemlyanoy Val Str. 73, Moscow 109004, Russia; v_p_s@mail.ru

**Keywords:** cardiolipin-coated nanoceria, oxidative metabolism genes, oxidative DNA damage, cell proliferation, autophagy, human embryonic lung fibroblasts

## Abstract

The unique redox properties of nanoscale cerium dioxide determine its diverse application in biology and medicine as a regulator of oxidative metabolism. Lipid modifiers of the nanoparticle surface change their biochemical properties and bioavailability. Complexes with lipids can be formed upon contact of the nanoparticles with the membrane. The effects of lipid coating on nanoceria have not been studied yet. Here, we assessed the effect of bare and cardiolipin-coated CeO_2_ on the expression of oxidative metabolism genes in human embryonic lung fibroblasts. Cell viability, mitochondrial activity, intracellular reactive oxygen species, NOX4, NRF2, and NF-κB expression, oxidative DNA damage/repair, autophagy, and cell proliferation were studied. We used an MTT assay, fluorescence microscopy, real-time reverse transcription polymerase chain reaction, and flow cytometry. At a concentration of 1.5 μM, bare and cardiolipin-coated nanoceria penetrated into cells within 1–3 h. Cell survival, mitochondrial activity, and the proliferative effect were similar for bare and cardiolipin-coated nanoceria. Intracellular ROS, activation of NOX4, NRF2, and NF-kB, DNA oxidative damage, and DNA break/repair were different. Cardiolipin-coated nanoceria induced intracellular oxidative stress and short-term activation of these genes and DNA damage/break/repair. Unlike bare nanoceria, cardiolipin-coated nanoceria induced autophagy. Thus, the effects of cardiolipin-coated nanoceria are determined by both the nanoceria itself and cardiolipin. Presumably, the differences in properties are due to lipid peroxidation of cardiolipin. This effect needs to be taken into account when developing nanoceria-based drugs targeting mitochondria.

## 1. Introduction

The key chemical feature of cerium dioxide is its ability to neutralize reactive oxygen species (ROS) due to the unique catalytic properties of its surface. Nanoceria not only acts as an ROS scavenger, but also exhibits enzyme-like (nanozyme) properties. The superoxide dismutase-like (SOD-like) activity of CeO_2_ was the first property to be discovered [1]. The SOD-like activity of nanoceria is the highest in comparison with the other SOD-mimetics and close to the activity of the native enzyme [2]. Its catalase- and peroxidase-like properties were studied by Pirmohamed et al. and Ivanov et al. [3,4]. Cerium dioxide nanoparticles also inactivate reactive nitrogen species [5,6] and exhibit oxidase- [7,8] and phosphatase-like activity [9]. Recently, new types of CeO_2_ biochemical activity have been discovered such as photolyase- [10], phospholipase- [11], and nuclease-like [12] properties. Cerium dioxide exhibits not only antioxidant but also hypoglycemic features [13]. All these features provide impressive prospects for nanoceria’s application in biology and medicine for modulating oxidative metabolism [14,15,16], including antibacterial, antioxidant, and anticancer therapy, drug/gene delivery systems, antidiabetic effects, and tissue engineering [17,18,19]. CeO_2_ nanoparticles have been successfully used as a nanoplatform for wound repair [20], as a protective agent against anesthesia-induced neurotoxicity and cognitive impairment [21], for increasing the radiosensitivity of tumors and protecting normal tissues from radiation damage [22], for treating cancer [23] and retinopathy [24], and for regenerating the nervous tissue in the treatment of the stroke and neurotrauma [25].

Surface modifiers are often used in the development of nanopharmaceuticals. These modifiers can act as stabilizers of colloidal solutions, facilitate the penetration of nanoparticles into cells, change the biochemical activity of nanoparticles, and reduce their toxicity. Bioavailability and biotransformability of inorganic nanoparticles are a weak point of their application. As for nano-CeO_2_, this problem is typically solved by using organic coatings [26,27] or by reducing the size for greater uptake by macrophages [28]. It is obvious that lipid coatings make the particles lipophilic and facilitate their penetration through membranes [29,30]. There are very few articles devoted to the study of lipid-coated nanoceria. Das et al. designed cationic lipid-nanoceria hybrids for gene delivery into mammalian cells [31]. Battaglini et al. designed a therapeutic system composed of biomimetic lipids and nanoceria, which has antioxidant properties and the ability to cross the blood–brain barrier [32].

Cardiolipin (CL) is an unsaturated phospholipid that is part of mitochondrial membranes. Cardiolipin liposomes were previously used for delivery of DNA and small interfering RNA in vitro and in vivo [33,34,35]. Cardiolipin was used as a drug delivery system across the blood–brain barrier [36]. The incorporation of cardiolipin into a polyurethane film resulted in improved biocompatibility towards blood vessels [37]. Cardiolipin nanodiscs including doxorubicin significantly reduced doxorubicin toxicity, maintaining its biological effectiveness [38]. Cardiolipin in prokaryotic cells plays a key role in the delivery of transcription factor decoys to bacteria [39]. Thus, as a surface modifier and a drug delivery system, cardiolipin deserves to be studied.

Theoretically, CeO_2_ nanoparticles can interact with membrane cardiolipin and form a complex with it. Modified with triphenylphosphine and ROS-responsive organic polymer, nanoceria can effectively neutralize mitochondrial ROS [40]. Liao et al. developed a nanoceria-based antioxidant system targeting mitochondria [41]. Yang et al. designed albumin–nanoceria nanoclusters with triphenyl phosphate, which have SOD-like activity in mitochondria for radiation protection [42]. Nanoceria conjugated with triphenylphosphonium can penetrate mitochondria [43,44]. Platinum-modified nanoceria are capable of selectively attaching to mitochondria [45]. Nanoceria modified with lipid self-assembling nanoparticles exhibited peroxidase- and SOD-like properties, protecting neuronal mitochondria from damage [46]. No articles devoted to studying the effect of cardiolipin-modified cerium dioxide on human genes and cells could be found in the literature.

Nanoparticles can enter the human body through the gastrointestinal tract, skin, and respiratory tract [47]. Though nanoceria is not yet used as a clinically approved medical product, it is already widely used in industry [48]. Hence, inhalation of CeO_2_ nanoparticles with air in the work area may pose a danger to humans. Exposure of nanoceria to the lungs can cause pneumonia, pneumoconiosis, and fibrosis [49]. The authors of the review note the need for further research into the mechanisms of action of nanoceria on the lungs. On the other hand, human embryonic lung fibroblasts are a convenient and widely used in vitro model for studying cyto- and geno-toxicity due to their rapid and stable response to exposure [50,51].

Here, we aimed to study the effects of cardiolipin-coated nano-sized cerium dioxide on the oxidative metabolism of human embryonic lung fibroblasts. We studied the following: (1) cell viability and mitochondrial activity, (2) intracellular oxidative stress, (3) expression of NOX4, NRF2, and NF-κB, (4) oxidative DNA damage, breaks and repair, (5) cell proliferation, and (6) autophagy. The results of our research for cardiolipin-coated nanoceria were compared with the data obtained for bare nanoceria.

## 2. Materials and Methods

### 2.1. Synthesis of Bare and Cardiolipin-Coated CeO_2_ Nanoparticles

**Figure 1:**
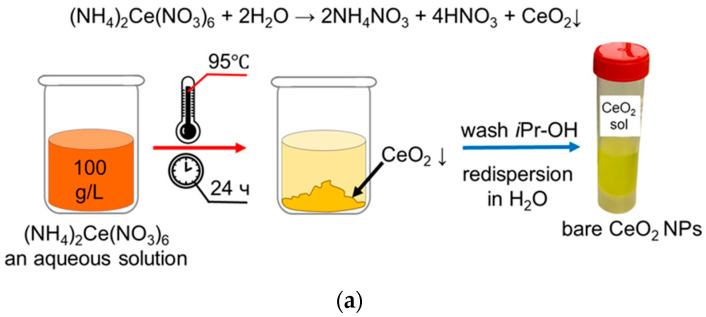

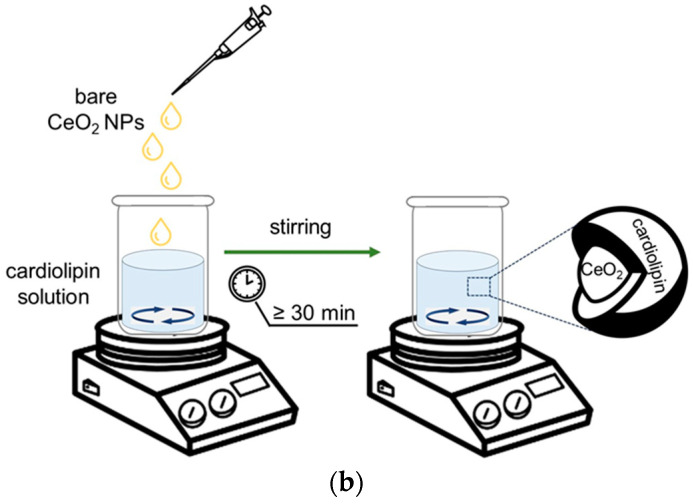
(**a**) Synthesis of bare CeO_2_ nanoparticles; (**b**) surface modification of CeO_2_ nanoparticles with cardiolipin at molar ratio 1:1 (50 μM).

Synthesis of an electrostatically stabilized colloidal solution of CeO_2_ was carried out using the thermal hydrolysis of ammonium cerium(IV) nitrate (#215473, Sigma, St. Louis, MO, USA) [52]. An aqueous solution of (NH_4_)_2_Ce(NO_3_)_6_ (100 g/L) was kept for 24 h in a drying oven at a temperature of 95 °C. The precipitate was separated from the mother liquor by centrifugation and then washed with isopropanol three times. The washed precipitate was redispersed in deionized water, followed by boiling for 1 h with constant stirring until complete removal of the isopropanol (T_boiling_ = 82.6 °C). A stock solution of cardiolipin (its structure is given in Supplementary Figure S1) (sodium salt from bovine heart, #C0563, Sigma, St. Louis, MO, USA) was prepared in a methanol/water mixture (4:1). The cardiolipin-coated CeO_2_ was obtained by gradual dropwise introduction of the stock CeO_2_ sol to the cardiolipin solution to a molar ratio of (1:1) with continuous stirring. After the addition of CeO_2_ was complete, the solution continued to be stirred for 30 min. As a result, cardiolipin is adsorbed onto the CeO_2_ nanoparticles. A scheme and a chemical equation illustrating the synthesis of cardiolipin coated nanoparticles are presented in Figure 1a,b.

### 2.2. Characterization of Cardiolipin-Coated Cerium Dioxide Nanoparticles

The concentration of the stock CeO_2_ sol was determined gravimetrically. Aliquots of the CeO_2_ sol were placed in previously brought crucibles to a constant mass and evaporated in a muffle furnace with subsequent heating to 900 °C. The samples were kept at this temperature for 4 h.

The microstructure of the bare CeO_2_ nanoparticles was analyzed by transmission electron microscopy on a Leo 912 AB Omega electron microscope (Carl Zeiss, Oberkochen, Germany) at an accelerating voltage of 100 kV. Before imaging, the sample was placed on a polymer-coated copper grid with a diameter of 3.05 mm. X-ray difermion analysis of the dried samples was performed using a Bruker D8 Advance diffractometer (Bruker, Billerica, MA, USA) (CuKα radiation, θ–2θ geometry) in the angular range of 3–120°2θ with a step of 0.01–0.02°2θ and a signal accumulation time of at least 0.3 s per point. Identification of diffraction maxima was carried out using the ICDD PDF2 database. Particle size was calculated using the Scherrer formula. 

The average hydrodynamic diameter of the CeO_2_ nanoparticles was estimated by dynamic light scattering using a Photocor Compact-Z analyzer (Photocor, Moscow, Russia) with the diode laser (λ = 650 nm, 25 mW). The measurements were performed at a scattering angle of 90° and room temperature. To measure zeta-potentials, a Nano ZS Zetasizer (Malvern Panalytical, Malvern, Worcestershire, UK) was used according to ISO/TR 19997:2018. For the analysis of the hydrodynamic diameters of the nanoparticles and zeta-potentials, 2 mL aqueous solutions with a CeO_2_ concentration of 1.5 mM were used.

UV-Vis spectra were recorded at room temperature using a Cary 4000 UV-Vis spectrophotometer (Agilent Technologies, Santa Clara, CA, USA) in 1.0 cm quartz cuvettes. Fluorescence spectra (λ_ex_ = 280 nm, slit width 5 nm) were recorded by a FluoroLog 3 spectrofluorometer (HORIBA Jobin Yvon SAS, Kyoto, Japan).

To assess the binding affinity of the ligand molecules to the surface of the CeO_2_ nanoparticles, attenuated total reflectance-Fourier transform infrared spectroscopy (ATR-FTIR spectroscopy) was used. The FTIR spectra were recorded using a VERTEX 70 Fourier spectrometer (Bruker, Billerica, MA, USA) with a diamond crystal on a GladiATR™ ATR attachment (PIKE Technologies, Madison, WI, USA). The FTIR spectra were recorded in the range from 4000 to 150 cm^−1^; the number of spectra and background scans was 64 with the resolution of 2 cm^−1^ and a crystal temperature of 50 °C. To record the spectra of the solutions, up to 5 μL of the sample was applied to the plate. The samples were allowed to dry completely for a duration of 4–5 min before proceeding with spectral analysis.

Fluorescence spectra were recorded on a Perkin Elmer LS 55 single-beam spectrometer (Perkin Elmer, Shelton, CT, USA). The excitation source was a 150 W xenon lamp operating in pulsed mode with a frequency of 50 Hz. A monochromator type of Monk-Gillison was employed in the wavelength range of 200–700 nm, with a spectral slit width of 10 nm for both excitation and emission. The scanning speed was 100 nm/min.

### 2.3. Cell Culture

Human embryonic lung fibroblasts (HELFs) (the 4th passage) were obtained from the Scientific Centre for Medical Genetics (Moscow, Russia). The cells were seeded at a concentration of 1.7 × 10^4^ cells/mL in Dulbecco’s modified Eagle’s medium (PanEco, Moscow, Russia) with 10% fetal calf serum (PAA, Vienna, Austria), 50 U/mL penicillin, 50 μg/mL streptomycin, and 10 μg/mL gentamicin. HELFs were cultured at 37 °C for 24 h, then cardiolipin-coated nanoceria was added. The cells were exposed to nanoparticles for 1, 3, 24, and 72 h.

### 2.4. MTT and TMRM Assays

The test with 3-(4,5-dimethylthiazol-2-yl)-2,5-diphenyltetrazolium bromide) (the MTT test) was applied to study cell viability. An EnSpire Plate Reader (EnSpire Equipment, Turku, Finland) was used to measure fluorescence at 550 nm. HELFs incubated with culture medium in deionized water were used as a negative control. As a positive control, incubation with dimethyl sulfoxide (0.0001–50%) was used as described elsewhere [53]. The cells were incubated with bare and cardiolipin-coated nanoceria for 72 h. The test with a membrane-voltage-dependent dye, tetramethylrhodamine methyl ester (TMRM) (Thermo Fisher, Waltham, MA, USA), was performed as described elsewhere [54].

### 2.5. Intracellular ROS Visualization

An AxioImagerA2 microscope (Carl Zeiss, Oberkochen, Germany) was used for fluorescent imaging. The cells were cultured in slide flasks and exposed to bare or cardiolipin-coated nanoceria. Each slide-bottom flask was seeded with approximately 500,000 cells. The medium was removed, the cells were washed with phosphate buffered saline (PBS), and dichloro-dihydro-fluorescein diacetate was added (a stock solution 2 mg/mL was diluted with PBS (1:200) before using). After incubation for 15 min, the cells were washed with PBS and immediately studied. No less than 100 fields of view were analyzed. The acquisition time was 6–10 s. Fluorescence intensity per cell and the total fluorescence were analyzed using the microscope software (ZEN 3.10).

### 2.6. Antibodies

Primary antibodies DyLight488-γH2AX (pSer139) (nb100-78356G, NovusBio, Centennial, CO, USA), FITC-NRF2, (bs1074r-fitc, Bioss Antibodies Inc., Woburn, MA, USA), FITC-BRCA1 (Nb100-598F, NovusBio, Centennial, CO, USA), PE-8-oxo-dG (sc-393871 PE, Santa Cruz Biotechnology, Dallas, TX, USA), CY5.5-NOX4 (bs-1091r-cy5-5, Bioss Antibodies Inc., Woburn, MA, USA), A350-BCL2 (bs-15533r-a350, Bioss Antibodies Inc., Woburn, MA, USA), NFKB (bs-0465r-cy7, Bioss Antibodies Inc., Woburn, MA, USA), LC3 (NB100-2220 NovusBio, Centennial, CO, USA), and PCNA (ab2426, Abcam plc, Cambridge, UK), and secondary anti-rabbit IgG-FITC (sc-2359, Santa Cruz Biotechnology, Dallas, TX, USA), were used.

### 2.7. Flow Cytometry

Flow cytometry of the unfixed cell suspension was used to determine intracellular ROS. The samples were incubated with 10 μM H2DCFH-DA (2,7-dichlorodihydrofluorescein diacetate) in PBS (Molecular Probes/Invitrogen, Carlsbad, CA, USA) for 15 min in the dark, washed with PBS, resuspended in PBS, and analyzed in the FITC (fluorescein isothiocyanate) channel (CytoFlex S, Beckman Coulter, Brea, CA, USA).

To quantify the content of proteins, HELFs were washed with Versene solution (Thermo Fisher Scientific, Waltham, MA, USA), treated with 0.25% trypsin (Paneco, Moscow, Russia), washed with the culture medium, and then suspended in PBS (pH 7.4) (Paneco, Moscow, Russia). Next, the cells were fixed with paraformaldehyde (Sigma-Aldrich, Saint Louis, MO, USA) at 37 °C for 10 min, washed three times with 0.5% BSA–PBS (BSA is bovine serum albumin), treated with 0.1% Triton X-100 in PBS for 15 min at 20 °C or with 90% methanol at 4 °C, and washed 3 times with 0.5% BSA–PBS. Next, the cells were stained with conjugated antibodies (1 μg/mL) for 2 h at room temperature, washed with PBS, and analyzed by a flow cytometer (CytoFlex S, Beckman Coulter, Brea, CA, USA).

### 2.8. Polymerase Chain Reaction Analysis for mRNA Quantitation

The mRNA was assessed with StepOnePlus (Applied Biosystems, Waltham, MA, USA) using *TBP* (TATA-box binding protein) as a reference gene. Total mRNA was isolated using the RNeasy Mini Kit (Qiagen, Hilden, Germany), treated with DNAse I, and then reverse transcribed by the Reverse Transcriptase kit (Sileks, Moscow, Russia). A quantitative reverse transcriptase polymerase chain reaction (qRT-PCR) method with SYBR Green PCR Master Mix (Applied Biosystems, Foster City, CA, USA) was applied for examining gene expression. The following primers were used (Sintol, Moscow, Russia): *NF-κB1* (F: CAGATGGCCCATACCTTCAAAT; R: CGGAAACGAAATCCTCTCTGTT); *NRF2* (NFE2L2) (F: TCCAGTCAGAAACCAGTGGAT, R: GAATGTCTGCGCCAAA AGCTG); *NOX4* (F: TTGGGGCTAGGATTGTGTCTA; R: GAGTGTTCGGCACATGGGTA); *BRCA1* (F: TGTGAGGCACCTGTGGTGA, R: CAGCTCCTGGCACTGGTAGAG); *LC3* (F: GCGAGTTACCTCCCGCAG; R: TCATGTTGACATGGTCCGGG); and *TBP* (reference gene) (F: GCCCGAAACGCCGAATAT, R: CCGTGGTTCGTGGCTCTCT).

To set up the reaction, a mixture of 5 μL RNA, 1 μL random hexaprimers, and 12 μL deionized water was placed in a thermostat at 70 °C for 5 min. This solution was then transferred to the cold for 10 min and 7 μL of a previously prepared solution (2.5 μL RT buffer, 4 μL 1.5 mM dNTP, and 0.5 μL M-MLV reverse transcriptase) was added. The resulting solution was kept at 25 °C for 11 min and then at 37 °C for 60 min. The composition of the PCR reaction mixture in a total volume of 25 μL is as follows: 10.35 μL deionized water, 6 μL Mg^2+^, 2.5 μL PCR buffer (700 mmol/L Tris-HCl, pH 8.6; 166 mmol/L ammonium sulfate; 35 mmol/L MgCl_2_), 2 μL 1.5 mmol/L dNTP solution, 0.75 μL DMSO (dimethyl sulfoxide), 1 μL of 30 pmol/L primer solution, 1 μL cDNA, 0.3 μL Tag polymerase (thermostable DNA polymerase I), 0.2 μL SybrGreen dye. The PCR annealing temperature was selected individually for each pair of primers. Standard PCR conditions were used: denaturation (95 °C for 4 min), then 40 amplification cycles (94 °C for 20 s, (56–62) °C for 30 s, 72 °C for 30 s), and then elongation (72 °C for 5 min). The gene expression level was analyzed in several (at least three) independent experiments. PCR conditions (and, above all, the amplification efficiency) of the standard series (*TBP*) are close to the PCR conditions of the experimental samples. We performed a direct comparison of the expression data of the studied genes using the StepOnePlus device software (StepOne™ Software v2.3) (Applied Biosystems, Waltham, MA, USA) and compared them with the method based on the calibration curve. The data were obtained using the device software (StepOne™ Software v2.3) and calculated by comparing concentrations based on the calibration curves (for an efficiency of at least 90%) coincide. Good reproducibility of the results was obtained in the series of experiments; the error was ~2%.

### 2.9. Chemiluminescence Assay for Lipid Peroxidation

A chemiluminescent method was used to analyze the lipid peroxidation of cardiolipin in the presence of cerium dioxide as described elsewhere [55,56]. Briefly, a coumarin 334 solution aliquot (50 µM, #393002, Sigma-Aldrich, Saint Louis, MO, USA) was added to a cuvette with a phosphate buffer solution (100 mM, pH 7.4). After recording the background signal for 30–60 s, an aliquot (50 µL) of the test sample was injected using a chromatography Hamilton syringe (Sigma-Aldrich, Saint Louis, MO, USA), without interrupting the chemiluminescence recording. This procedure allowed for the rapid recording of the kinetics of the peroxidation processes. Registration of the kinetics was performed for 10 min. As an analytical signal, the light sum was used, which is the area under the chemiluminescence curve over a certain time interval (5 min) and is directly proportional to the number of formed radicals.

The intensity of chemiluminescence was measured at room temperature on a 1-channel Lum-100 chemiluminometer (DISoft, Moscow, Russia). All experiments were conducted in triplicate. The results were processed using the PowerGraph software (version 3.3) (DISoft, Moscow, Russia).

### 2.10. Statistics

Three parallel measurements were performed for each experiment. The data are presented as mean and standard deviation. Differences were considered significant at *p* < 0.01 (the non-parametric Mann–Whitney test). The StatPlus2007 Pro v4.9.2 software (AnalystSoft Inc., Walnut, CA, USA) was used for statistical analysis.

## 3. Results

### 3.1. Cardiolipin-Coated CeO_2_ Nanoparticles

#### 3.1.1. Synthesis and Characterization of Size and Surface Potential

An electrostatically stabilized aqueous CeO_2_ sol was obtained by thermal hydrolysis of (NH_4_)_2_Ce(NO_3_)_6_. The concentration of the CeO_2_ sol was 0.130 ± 0.004 mol/L (22.3 g/L).

Table 1 shows the zeta potentials and particle sizes of bare and cardiolipin-coated cerium dioxide nanoparticles determined with powder X-ray diffraction and dynamic light scattering.

Powder X-ray diffraction showed that the CeO_2_ sols contain single-phase fluorite-type cerium dioxide (PDF2 34-0394). Cardiolipin coating did not change the X-ray diffraction patterns or the positions of the peaks (111), (200), (220), and (311) (see Supplementary Figure S2c).

The hydrodynamic diameters estimated by dynamic light scattering were three times larger than the sizes estimated with X-ray diffraction due to the mild aggregation of bare CeO_2_ nanoparticles in aqueous solutions (see Supplementary Figure S5a). Modification of cerium dioxide with cardiolipin results in the recharging of the surface of nanoparticles, leading to the formation of a predominantly 38 ± 5 nm particle fraction (see Supplementary Figure S5b). Polydispersity index (PDI) values were calculated for the ceria sols. PDI values of 0.006 for bare CeO_2_ nanoparticles and 0.02 for cardiolipin-coated CeO_2_ nanoparticles indicate that both types of samples exhibit significant monodispersity.

The ζ-potential for bare CeO_2_ of +39.6 ± 0.3 mV indicates the high stability of the aqueous sol. Interaction of the CeO_2_ nanoparticles with cardiolipin led to the surface recharge due to the binding of the CeO_2_ nanoparticles to the phosphate groups of cardiolipin. A high zeta potential value (ζ = −36.6 ± 0.4) also indicates the stability of the cardiolipin-coated nanoceria sol.

The particle sizes and phase composition of the synthesized materials were additionally proved by transmission electron microscopy and electron diffraction (see Supplementary Figure S2a,b). The images show that the nanoparticles form aggregates of up to 10–15 nm in size when dried for TEM imaging. Figure S2b demonstrates that CeO_2_ nanoparticles formed denser aggregates in the presence of a lipid ligand.

Using calculations, we estimated that a molar ratio of ligand to cerium dioxide of 0.07:1 is required for complete coating of the nanoparticles with cardiolipin (see Supplementary). Thus, we can assume that at a ratio of 1:1 (one and a half orders of magnitude excess in concentration) nanoceria is completely covered with cardiolipin.

#### 3.1.2. Spectral Characterization

Electronic absorption spectra of bare and CL-coated CeO_2_ contain a characteristic absorption band in the region of 280–300 nm (see Supplementary Figure S4). Modification of the surface of the CeO_2_ nanoparticles did not lead to any changes in their characteristics.

The spectrum of the colloidal solution of bare cerium dioxide nanoparticles is characterized by a high fluorescence intensity with a maximum at 356 nm (See Supplementary Figure S6). For cardiolipin-coated nanoceria, a poorly resolved peak with lower intensity is observed in the fluorescence spectrum with the maxima at 356 nm and 448 nm (Figure S6). The lower intensity can be explained by the quenching effect of cardiolipin [57], while the additional peak can be associated with the contribution of cardiolipin fluorescence. This further indicates that the modification of CeO_2_ nanoparticles by the ligand has indeed occurred.

To assess the binding affinity of the ligand molecules to the surface of the CeO_2_ nanoparticles, ATR-FTIR spectroscopy was used. In the FTIR spectrum of bare CeO_2_ (see Supplementary Figure S3), the primary absorption band at 470 cm^−1^ corresponds to the stretching vibrations of the Ce–O bond [58]. The absorption band at 1624 cm^−1^ was primarily due to H–O–H vibrations [59]. The absorption band at 1289 cm^−1^, as well as the unresolved peak with maxima at 1512 cm^−1^ and 1472 cm^−1^, may correspond to residual nitrate ions from the synthesis precursor (NH_4_)_2_Ce(NO_3_)_6_ [60]. The bands that appeared at 1034 cm^−1^, 809 cm^−1^, and 740 cm^−1^ are due to presence of organic components (the bending C–OH and stretching C–O vibrations of isopropanol traces) [61,62]. The FTIR spectrum of cardiolipin (see Supplementary Figure S3) was characterized by absorption bands at 1738 cm^−1^ (ν(C=O)), 1555 cm^−1^ and 1460 cm^−1^ (δ(C–H)), 1378 cm^−1^ (δ(–CH_3_)), 1175 cm^−1^ and 1095 cm^−1^ (ν(P–O)), 1067 cm^−1^ and 877 cm^−1^ (δ(C–OH) and δ(P–O)), 726 cm^−1^ (δ(C–C)), and 604 cm^−1^ (δ(P–O–P)) [63]. The FTIR spectrum of the cardiolipin-coated CeO_2_ sol (see Supplementary Figure S3) showed shifts of the Ce–O bond bands from 470 cm^−1^ to 495 cm^−1^, which confirmed the presence of CeO_2_ and its bond with organic groups. The FTIR spectrum also showed a shift of the characteristic bands at 1175 cm^−1^ and 1067 cm^−1^ for P–O to 1195 cm^−1^ and 1055 cm^−1^, respectively. Such shifts indicate the formation of complexes between the phospholipid molecule and CeO_2_ nanoparticles through the formation of coordination bonds or non-covalent hydrogen bonds.

#### 3.1.3. Cardiolipin Peroxidation

**Figure 2:**
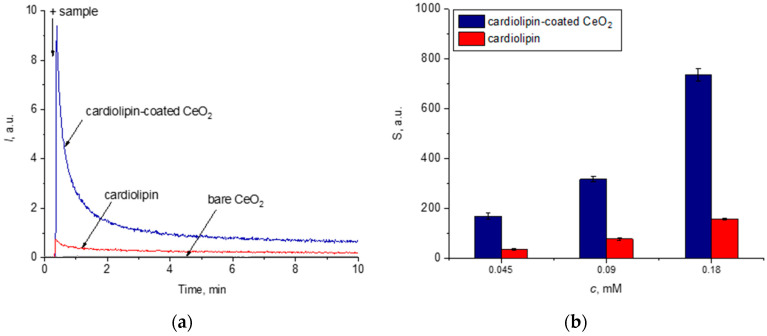
(**a**) Chemiluminograms of the bare CeO_2_ nanoparticles (0.18 mM), cardiolipin solution (0.18 mM), and cardiolipin-coated CeO_2_ nanoparticles (1:1, 0.18 mM) in a phosphate buffer solution (100 mM, pH 7.4) with coumarin 334 (50 μM) and (**b**) histograms of the light sums for the ceria sols and cardiolipin solution.

Lipid peroxidation of cardiolipin in the complex with nanoscale cerium dioxide was assessed using the chemiluminescence protocol. As a selective enhancer to (phospho)lipid radicals, coumarin 334, was applied. Figure 2a shows chemiluminograms recorded upon the addition of the samples to the reaction mixture containing coumarin 334 and phosphate buffer solution. The addition of various concentrations of cardiolipin and cardiolipin-coated CeO_2_ nanoparticles to the reaction mixture resulted in a dose-dependent enhancement of chemiluminescence (Figure 2b). The increase in chemiluminescence compared to the control level indicates the presence of lipid peroxidation products, in particular lipid hydroperoxides. Notably, after the immobilization of cardiolipin molecules on the surface of cerium dioxide nanoparticles, the degree of lipid oxidation increased by four times. This is consistent with previously obtained data confirming that CeO_2_ nanoparticles possess lipo- and phospholipo-peroxidase-like activities [55].

We consider these results as a basis for future studies on peroxidation of phospholipids in complex with nanoceria. However, these data convincingly indicate the phenomenon of cardiolipin peroxidation in the presence of nanoceria, which can explain the differences in the behavior of bare and cardiolipin-coated nanoceria in relation to oxidative metabolism genes.

### 3.2. Cell Viability

To access cell viability, we performed a 72-h MTT test as relative absorbance at 570 nm vs. concentration of CeO_2_. Though the applicability of this assay to nanoparticles has not yet been fully explored because of a complex mechanism of interaction between the nanoparticles and MTT, it is widely used to assess cell survival especially when comparing different nanoparticles [64,65,66,67,68], including nanoceria [69,70]. As we intended to compare bare and cardiolipin-coated nanoceria and to select a safe concentration for the cell-based experiments, we considered it possible to use an MTT assay in our study.

**Figure 3:**
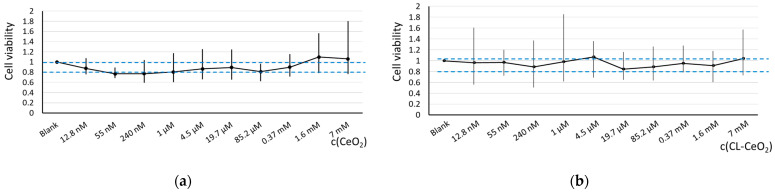
The 72-h MTT test for: (**a**) bare CeO_2_ and (**b**) cardiolipin-coated CeO_2_; in blank experiments, cells were incubated without nanoceria. Blue dashed lines indicate a viability range (0.8–1.0).

Both bare and CL-coated nanoceria showed no toxicity up to a 7 mM concentration, though incubation with bare CeO_2_ in the 55 nm—1 µM range resulted in viability slightly lower than 0.8 (Figure 3a). Cardiolipin coating makes the nanoceria substance less toxic towards HELFs (Figure 3b). A concentration of 1.5 μM for bare and CL-coated CeO_2_ was chosen for all the following experiments.

### 3.3. Mitochondrial Potential as Determined with the TMRM Assay

**Figure 4:**
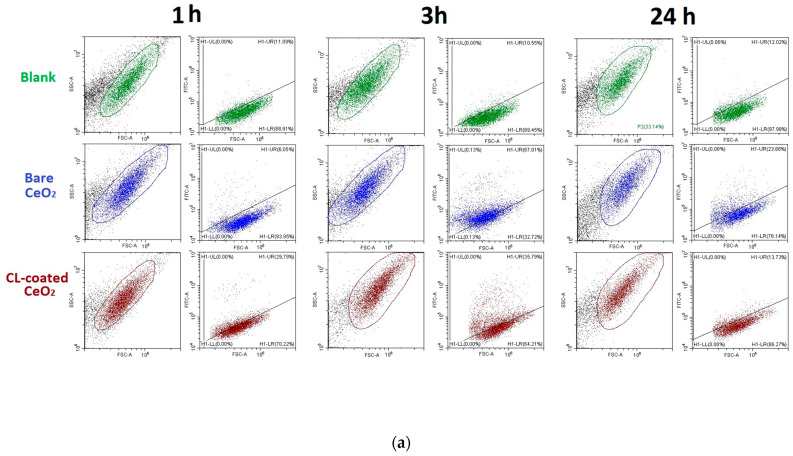

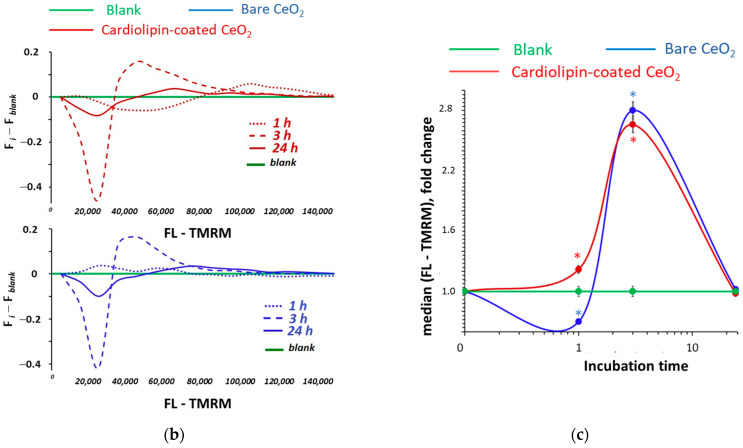
The TMRM test using flow cytometry: (**a**) the SSC vs. FSC dot plot (the left panels) and fluorescence of TMRM vs. FSC in the selected regions (the right panels) for 1, 3, and 24 h of exposure; (**b**) the difference in TMRM fluorescence for cells incubated with the test compounds and the control cells; and (**c**) the relative enhancement of the TMRM signal in cells relative to the blank (medians) vs. incubation time for 1.5 μM bare and cardiolipin-coated (CL-coated) nanoceria. In blank experiments, cells were incubated without nanoceria. The significant difference according to the Mann–Whitney test (*p* < 0.05) is marked with *.

The TMRM (tetramethylrhodamine methyl ester) test was performed using flow cytometry. TMRM is a cell-permeant, fluorescent dye that is readily sequestered by active mitochondria. Figure 4 presents the results of the TMRM test as a function of incubation time (Figure 4b,c), as well as the flow cytometry data for all incubation time points (Figure 4a). For bare nanoceria, mitochondrial activity fell within the first hour, then increased within three hours. After 24 h of incubation, the fluorescence decreased to the control values (Figure 4). For CL-coated CeO_2_, the TMRM fluorescence increased after 1–3 h of exposure. Within 24 h, the effects of bare and cardiolipin-coated nanoceria were the same. Interestingly, cardiolipin-coated nanoceria does not have a suppressive effect on mitochondrial activity. This may be due to the presence of cardiolipin on the nanoparticle surface.

### 3.4. Penetration into Cells

**Figure 5:**
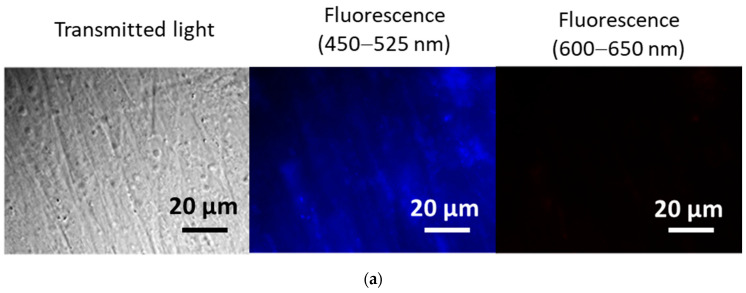

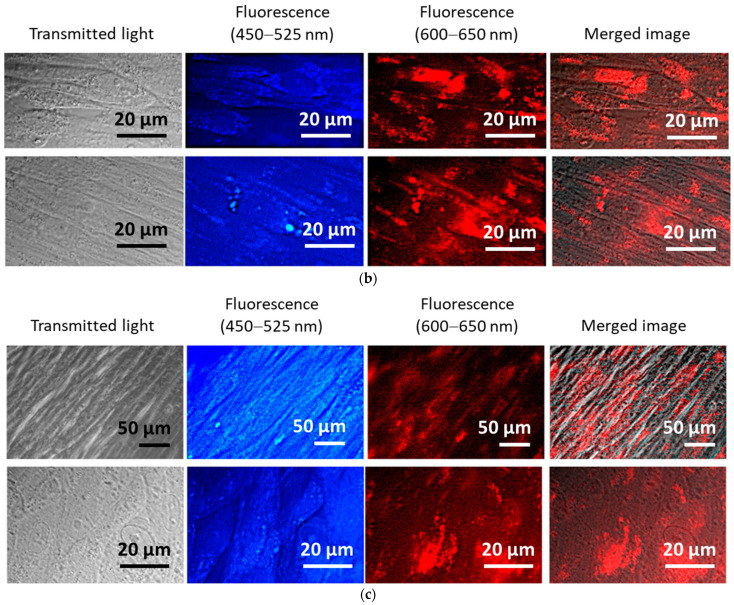
Transmitted light and fluorescence images of human embryonic lung fibroblasts; (**a**) control cells; (**b**) bare nanoceria (1.5 μM) in cells after 3 h of incubation; and (**c**) cardiolipin-coated nanoceria (1.5 μM) in cells after 3 h of incubation. Excitation wavelength, 370 nm. Magnification, 100×.

The fluorescence spectra of bare and cardiolipin-coated nanoceria showed intrinsic fluorescence in a wide band of 325–450 nm (the spectra are given in Supplementary Figure S6). Control cells did not exhibit autofluorescence (Figure 5a). Exposure of the cells to bare and cardiolipin-coated nanoceria resulted in autofluorescence (Figure 5b,c). Thus, during the first three hours, bare and cardiolipin-coated nanoceria penetrate the cells.

### 3.5. Intracellular ROS

**Figure 6:**
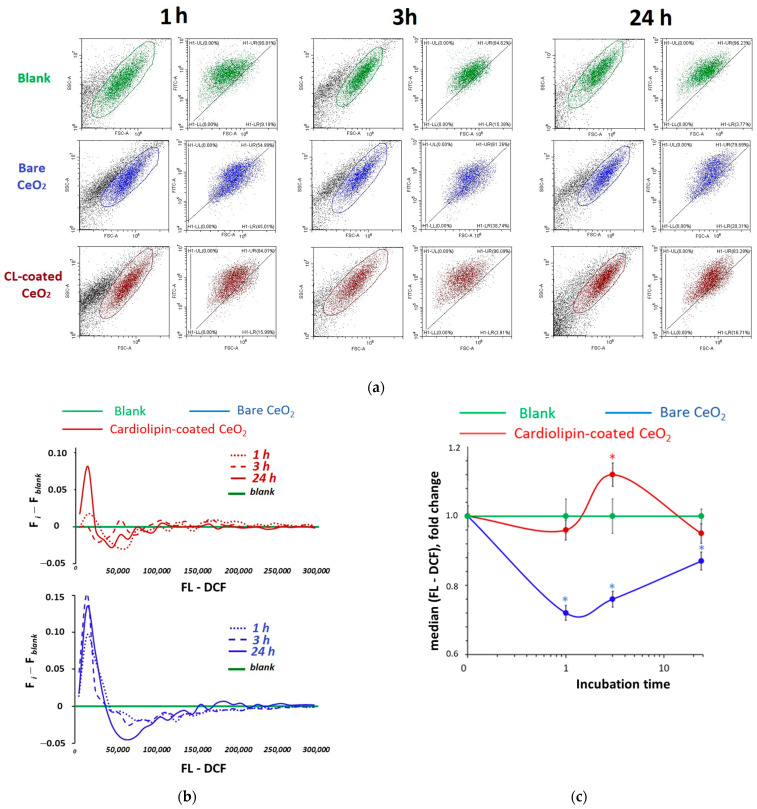
Intracellular ROS assessed with flow cytometry: (**a**) the SSC vs. FSC dot plot (the left panels) and fluorescence of DCF vs. FSC in the selected regions (the right panels) for 1, 3, and 24 h of exposure; (**b**) the difference in DCF fluorescence for cells incubated with the test compounds and the control cells; and (**c**) the relative enhancement of the DCF signal in cells relative to the blank (medians) vs. incubation time for 1.5 μM of bare and cardiolipin-coated (CL-coated) nanoceria. In blank experiments, cells were incubated without nanoceria. The significant difference according to the Mann–Whitney test (*p* < 0.05) is marked with *.

Human embryonic lung fibroblasts were exposed to 1.5 μM of bare and cardiolipin-coated nanoceria for 1, 3, 24, and 72 h. An H2DCF-DA (2′,7′-dichlorodihydrofluorescein diacetate) assay was applied to examine the ROS content in the cells. H2DCF-DA penetrates through cell membranes and turns to non-fluorescent DCFH, which is oxidized by ROS (mostly H_2_O_2_) to fluorescent DCF. Figure 6 presents the results of intracellular ROS assessment as a function of incubation time (Figure 6b,c), as well as the flow cytometry data for all incubation time points (Figure 6a). The incubation of HELFs with cardiolipin-coated nanoceria to cells resulted in a slight decrease in the intracellular ROS level after 1 h, followed by an increase in the ROS level after 3 h, and normalization of the oxidative balance within 24 h (Figure 6). On the contrary, the addition of bare nanoceria (1.5 µM) to the cells results in a significant decrease in the intracellular ROS level after 24 h (Figure 6). 

Thus, bare nanoceria added to cells during 1–24 h act as ROS scavengers, while CL-coated nanoceria induce intracellular oxidative stress.

### 3.6. NOX4 Expression

**Figure 7:**
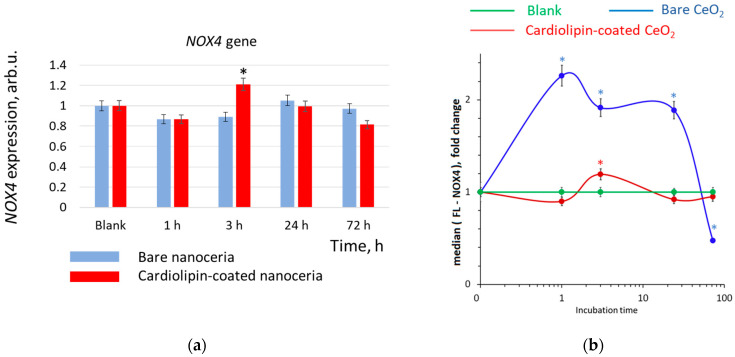
(**a**) *NOX4* gene and (**b**) NOX4 protein expression in human embryonic lung fibroblasts after exposure with bare and cardiolipin-coated nanoceria (1.5 μM) within 1–72 h. In blank experiments, cells were incubated without nanoceria. The *TBP* gene was used as an internal reference gene, the mean RNA amount was calculated from three experiments relative to control, and the significant difference according to the Mann–Whitney test (*p* < 0.05) is marked with *.

NADPH oxidase (NOX4) is a major source of superoxide anion radicals in cells. The effect of bare nanoceria causes a significant activation of NOX4 protein expression for 24 h, then a drop below the control level after 72 h (Figure 7b), while changes in *NOX4* gene expression are not significant (Figure 7a). Presumably, such activation of NOX4 is a response to intracellular ROS deficiency (Figure 6b). As for cardiolipin-coated nanoceria, the dynamics of NOX4 protein expression are different; they virtually repeat the dynamics of the intracellular ROS level (compare Figure 6b and Figure 7b). After an insignificant decrease in NOX4 level during the first hour, the NOX4 expression increases within 3 h and then decreases to the control level.

### 3.7. NRF2 Expression

**Figure 8:**
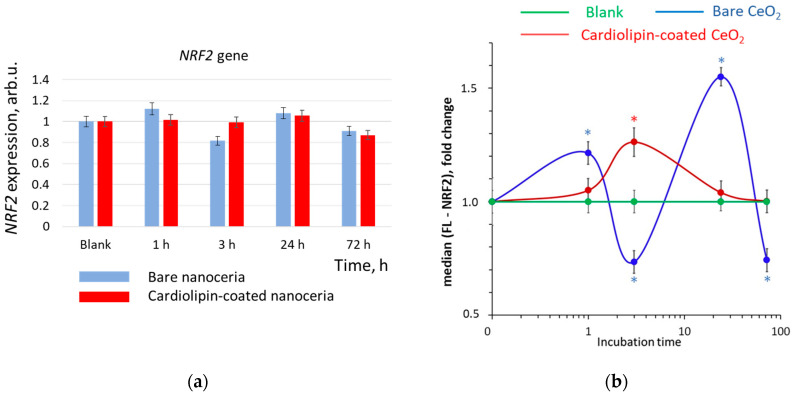
(**a**) *NRF2* gene and (**b**) NRF2 protein expression in human embryonic lung fibroblasts after exposure with bare and cardiolipin-coated nanoceria (1.5 μM) within 1–72 h. In blank experiments, cells were incubated without nanoceria. The *TBP* gene was used as an internal reference gene, the mean RNA amount was calculated from three experiments relative to control, and the significant difference according to the Mann–Whitney test (*p* < 0.05) is marked with *.

The NRF2 (nuclear factor erythroid-2-related factor 2) transcription factor is involved in a key pathway of the antioxidant response. For bare and cardiolipin-coated nanoceria, the changes in gene expression were not significant (Figure 8a). After incubation with bare nanoceria, the protein expression showed similar wave-like dynamics, but the changes were statistically significant (Figure 8b). Within an hour, the NRF2 protein expression increased, then fell below the control level, then increased again after 24 h, and fell below the control level after 72 h. For cardiolipin-coated nanoceria, the dynamics were fundamentally different. The NRF2 protein expression increased monotonically during 3 h of incubation, then fell to the control level after 72 h.

### 3.8. NF-κB Expression

**Figure 9:**
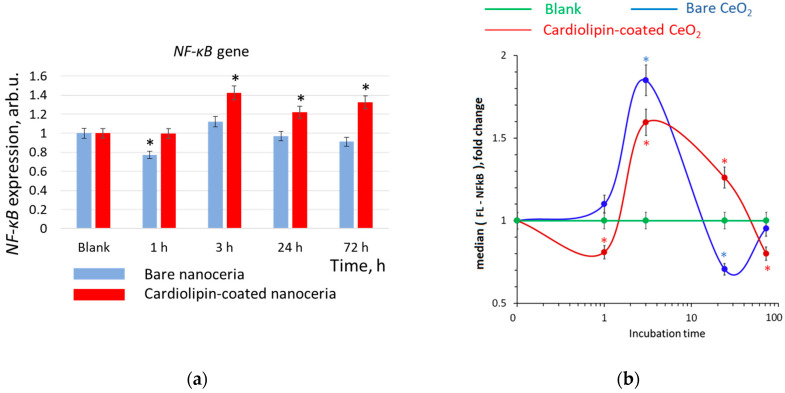
(**a**) *NF-κB* gene and (**b**) NF-κB protein expression in human embryonic lung fibroblasts after exposure with bare and cardiolipin-coated nanoceria (1.5 μM) within 1–72 h. In blank experiments, cells were incubated without nanoceria. The *TBP* gene was used as an internal reference gene, the mean RNA amount was calculated from three experiments relative to control, and the significant difference according to the Mann–Whitney test (*p* < 0.05) is marked with *.

The NF-κB (nuclear factor kappa-light-chain-enhancer of activated B cells) signaling pathway is involved in inflammation, cell proliferation, and apoptosis. The transcriptional activity of the *NF-κB* gene is usually inversely related to the activity of the *NFR2* gene. For bare nanoceria, the expression of the NF-κB transcription factor increased within 3 h, then fell below the control level after 24 h, and returned to the control level after 72 h (Figure 9b). Changes in gene expression were not significant (Figure 9a). For cardiolipin-coated nanoceria, the expression of the NF-κB transcription factor fell below the control level within 1 h, then increased after 3 h, and monotonically decreased below the control level after 72 h. *NF-κB* gene expression increased after 3, 24 and 72 h of incubation (Figure 9).

### 3.9. DNA Oxidative Damage and Double-Strand Breaks

**Figure 10:**
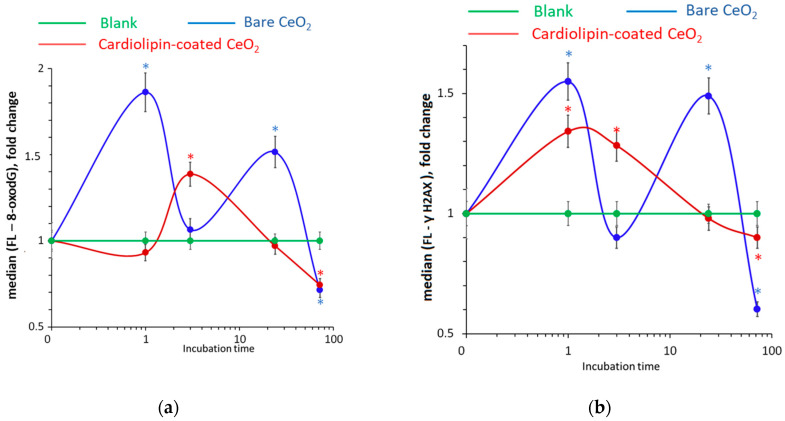
(**a**) 8-Oxo-2′-deoxyguanosine concentration and (**b**) phosphorylated histones γH2AX as biomarkers of oxidative damage and double-strand breaks relative to the control (the cells with no nanoceria added) as quantified by flow cytometry as a result of incubation of human embryonic lung fibroblasts with bare and cardiolipin-coated nanoceria (1.5 μM) within 1–72 h. The significant difference according to the Mann–Whitney test (*p* < 0.05) is marked with *.

8-Oxo-2′-deoxyguanosine (8-oxo-dG) is a marker of oxidative DNA damage. After 1 h of incubation with bare nanoceria, the content of 8-oxo-dG increased by 1.6–1.8 times. After 3 h, the content of 8-oxo-dG decreased to the control value, then increased, and decreased after 72 h to below the control value (Figure 10a). We examined DNA double-strand breaks using the γH2AX (H2A histone family member X) assay. After 1 h of exposure to bare nanoceria, phosphorylated γH2AX increased by 1.5 times, which correlated with the increase in 8-oxo-dG. After 3 h, the level of double-strand breaks decreased. After 24 h, it increased, and it decreased below the control values within 72 h (Figure 10b). In general, the “oscillatory” dynamics of changes in 8-oxo-dG and γH2AX are similar and corresponds to the dynamics of changes in NOX4 and NRF2.

For cardiolipin-coated nanoceria, the dynamics of oxidative DNA damage also corresponded to the dynamics of changes in NOX4 and NRF2. Within the first hour, there was a slight decrease in the level of 8-oxo-dG, then an increase after 3 h, and then a decrease below the control level after 72 h (Figure 10a). The level of double-strand damage increased within 1 h and then decreased monotonically to level below the control value within 72 h (Figure 10b).

Thus, for bare nanoceria, there is a “bright interval” within 3 h of incubation, when oxidative damage and breaks are practically negligible, while for cardiolipin-coated nanoceria, maximum oxidative damage and double-strand DNA breaks are achieved during 3 h of incubation.

### 3.10. DNA Repair

**Figure 11:**
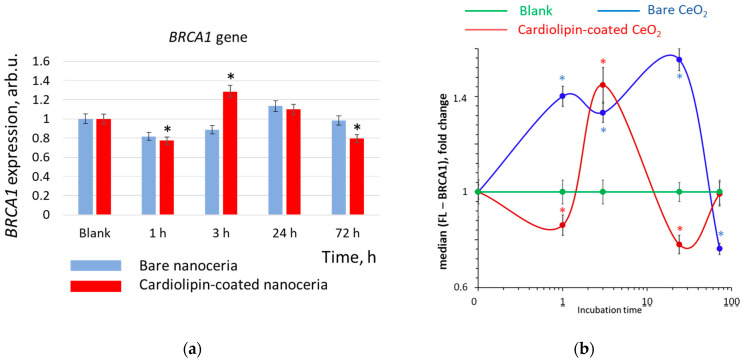
(**a**) *BRCA1* gene and (**b**) BRCA1 protein expression as a result of incubation of human embryonic lung fibroblasts with bare and cardiolipin-coated nanoceria (1.5 μM) within 1–72 h. In blank experiments, cells were incubated without nanoceria. The *TBP* gene was used as an internal reference gene, the mean RNA amount was calculated from three experiments relative to control, and the significant difference according to the Mann−Whitney test (*p* < 0.05) is marked with *.

DNA damage and breaks cause the activation of the repair system. The *BRCA1* (BReast CAncer 1) gene is a key repair gene. For the bare nanoceria, a decrease in the expression of the *BRCA1* gene, followed by its activation, was observed. However, the changes were not statistically significant (Figure 11a). Changes in the protein expression had the same oscillatory dynamics, but the changes were significant. After 1 h, the expression of the BRCA1 protein increased by 1.5 times (Figure 11b). The increased expression of the BRCA1 protein lasts for up to 24 h, and then it decreased below the control value.

The dynamics of the DNA repair system were different for cardiolipin-coated nanoceria. The maximum activation was observed within three hours of incubation (Figure 11b), which corresponds to the maximum of oxidative damage and breaks (Figure 10). After 1 h and 24 h of incubation, the activity of the repair system was decreased compared to the control. After 72 h, the activity of the repair system returned to the control level.

Thus, the effects of bare and cardiolipin-coated nanoceria are different with respect to oxidative DNA damage/breaks and activation of the DNA repair system.

### 3.11. Autophagy

**Figure 12:**
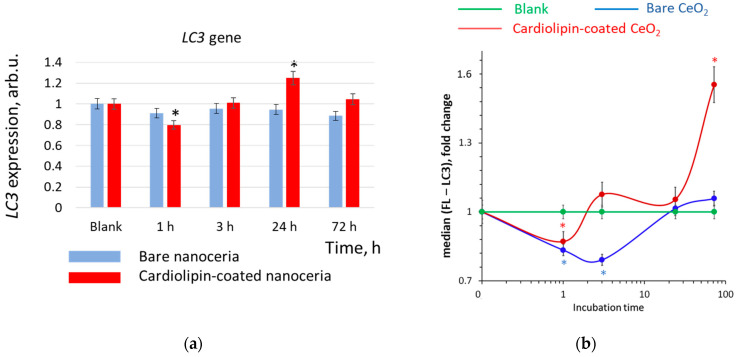
(**a**) *LC3* gene and (**b**) LC3 protein expression as a result of incubation of human embryonic lung fibroblasts with bare and cardiolipin-coated nanoceria (1.5 μM) within 1–72 h. In blank experiments, cells were incubated without nanoceria. The *TBP* gene was used as an internal reference gene, the mean RNA amount was calculated from three experiments relative to control, and the significant difference according to the Mann−Whitney test (*p* < 0.05) is marked with *.

The LC3 protein (microtubule-associated protein 1A/1B-light chain 3) is an autophagy marker protein associated with autophagosomes. The *LC3* gene expression in cells exposed to bare and CL-coated nanoceria had similar dynamics (Figure 12a). For cardiolipin-coated nanoceria, gene expression decreased by 20% within 1 h, then increased after 24 h, and decreased to the control level after 72 h. The changes in gene expression for bare nanoceria are not statistically significant. The LC3 protein expression also differs for bare and CL-coated nanoceria. Bare nanoceria (1.5 µM) caused a significant decrease in the expression of the LC3 autophagy protein by approximately 40% within 3 h (Figure 12b). After 24 and 72 h, the protein expression does not differ from the control. In contrast, when exposed to CL-coated CeO_2_, LC3 protein expression in the cells decreases in the first hour, but by 72 h increases markedly.

**Figure 13:**
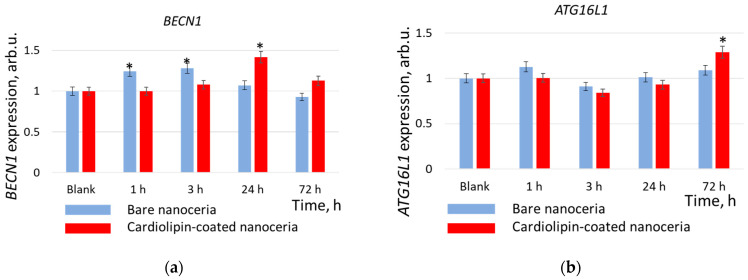
(**a**) *BECLN1* gene and (**b**) *ATG16L1* gene expression as a result of incubation of human embryonic lung fibroblasts with bare and cardiolipin-coated nanoceria (1.5 μM) within 1–72 h. In blank experiments, cells were incubated without nanoceria. The *TBP* gene was used as an internal reference gene, the mean RNA amount was calculated from three experiments relative to control, and the significant difference according to the Mann−Whitney test (*p* < 0.05) is marked with *.

The BECLN1 gene encodes the Beclin-1 protein which plays a key role in autophagy regulation [71]. The ATG16L1 gene encodes the autophagy related 16 like 1 protein [72], which is a subunit of the autophagy-related ATG12−ATG5/ATG16 complex and is essentially important for the lipidation of LC3 and autophagosome formation. Figure 13 shows the expression of the *BECLN1* and *ATG16L1* genes in HELFs cells. For bare nanoceria, *BECLN1* gene expression increased by a factor of 1.2 after 1 and 3 h of incubation, then decreased to the control level (Figure 13a). For cardiolipin-coated nanoceria, *BECLN1* gene expression increased by a factor of 1.4 after 24 h, then decreased. For cardiolipin-coated nanoceria, *ATG16L1* gene expression slightly decreases by about 20% within 3 h, then increases after 72 h by a factor of 1.3 (Figure 13b).

Thus, after 72 h, the inhibitory effect of bare nanoceria on autophagy had already disappeared, while the activation effect of CL-coated nanoceria remained high.

### 3.12. Cell Proliferation

**Figure 14:**
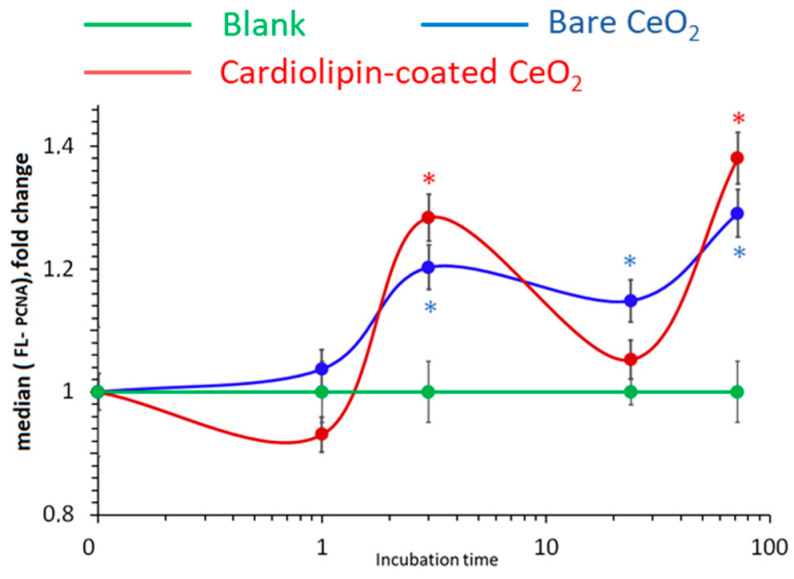
Cell number vs. PCNA fluorescence in human embryonic lung fibroblasts relative to the blank (medians) vs. incubation time for 1.5 μM bare and citrate-coated nanoceria. In blank experiments, cells were incubated without nanoceria. The significant difference according to the Mann−Whitney test (*p* < 0.05) is marked with *.

The exposure of HELFs to bare and CL-coated nanoceria led to the same wave-like effect. There was an increase in the expression of the PCNA (proliferating cell nuclear antigen) proliferation protein within 3 h of incubation, followed by a decrease within 24 h, and increase within 72 h by approximately 30–40% (Figure 14).

Thus, both bare and cardiolipin-coated nanoceria exhibit similar proliferative effects.

## 4. Discussion

The main results of the study can be summarized as follows (Table 2): (1) unlike bare nanoceria, which exhibits strong antioxidant properties inside human embryonic lung fibroblasts, CL-coated nanoceria causes short-term (within 3 h) oxidative stress; (2) unlike the “oscillating” and long-term effects of bare nanoceria on NOX4 and NRF2 and DNA damage/breaks and repair, CL-coated nanoceria has a short-term activating effect; (3) unlike bare nanoceria, CL-coated nanoceria activates autophagy within 72 h; and (4) both bare and CL-coated nanoceria have similar proliferative effects on the cells. To summarize, cardiolipin is not an indifferent component as it modifies the effects of nanoceria on oxidative metabolism genes and DNA damage, as well as autophagy.

Cerium dioxide nanoparticles were modified by adding a cardiolipin solution. The evidence of modification is a change in the zeta potential and surface charge. According to transmission electron microscopy, cardiolipin-modified nanoceria forms aggregates of approximately 10–15 nm in size. The DLS data indicate that the diameter of cardiolipin-coated nanoceria (38 ± 5 nm) is 3 times larger than that of bare nanoceria, which is due to the cardiolipin shell. Nanoparticles of this size can penetrate into the cell by translocating directly into the cytosol without endocytosis [45]. Penetration of the membrane by the nanoparticles was confirmed by autofluorescence of the cells (Figure 4).

The experiments demonstrate a rapid stimulation of NOX4, NRF2, and BRCA1 protein expression in cells exposed to bare nanoceria (see Figure 7b, Figure 8b and Figure 11b). On the one hand, this may be due to rapid activation of genes in the first 30 min, which was demonstrated elsewhere [73]. On the other hand, the rapid increase in NRF2 protein concentration can be explained by its release from the complex with KEAP1 (Kelch-like ECH-associated protein). Nanoscale cerium dioxide may cause activation of NADPH oxidase on the outer membrane [74]. Our own studies confirm the involvement of nanoceria in the direct activation of the neutrophil NADPH oxidase [75]. The second peaks of protein expression are associated with activation of transcription and translation and synthesis of new NRF2 and NOX4. Rapid activation of the NOX4 protein leads to increased oxidative DNA damage and double-strand breaks (Figure 10), which activate the repair system (Figure 11). In general, the phenomenon of rapid activation by bare nanoceria is interesting and requires a separate study at short exposure times (less than 60 min).

Our experiments have shown that there are similarities and differences in the effects of bare and cardiolipin-coated nanoceria on oxidative metabolism. The effects of bare and CL-coated nanoceria on cell survival and mitochondrial activity are similar, as well as the effects on cell proliferation. Both nano-CeO_2_ substances have proliferative effects, which is in agreement with the literature data [76,77,78].

The fundamental difference is in the effect of bare and CL-coated nanoceria on intracellular ROS. While bare nanoceria exhibits pronounced antioxidant properties with respect to hydrogen peroxide, which corresponds to the literature data [79,80], cardiolipin-coated nanoceria, on the contrary, causes oxidative stress. Apparently, this is due to the cardiolipin. The cardiolipin molecule contains four fatty acid tails and two orthophosphoric acid residues. In most animal tissues, cardiolipin contains C18 chains with two unsaturated bonds in each of them. The unsaturation of the alkyl chains results in the participation of cardiolipin in lipid peroxidation. Oxidized cardiolipin is capable of forming complexes and nanostructures with cytochrome C, which leads to the appearance of phospholipid peroxidase activity in cytochrome C and the induction of the mitochondrial apoptosis pathway [81,82]. Cytochrome C-bound cardiolipin is involved in lipid peroxidation. We can assume that nanoceria-bound cardiolipin is also involved in lipid peroxidation, which results in lipid oxidative stress. We have previously shown that nanoceria exhibits phospholipid peroxidase-like activity toward phosphatidylcholine hydroperoxide [55]. Our own preliminary data support the peroxidation of cardiolipin with nanoceria (Figure 2). Presumably, the mechanism of phospholipid oxidation is similar to the mechanism of lipid peroxidation, and the high phospholipid peroxidase-like activity of CeO_2_ nanoparticles was due to their affinity for phospholipids. Hypothetically, intracellular lipid oxidative stress changes ROS balance towards oxidative stress, as supported by Figure 6b. Since nanoceria has phosphatase activity [9,83,84], it is possible that dephosphorylation of cardiolipin may occur, which could affect phosphorous metabolism, cellular regulation, and signaling. In general, the mechanisms of interaction between nanoceria and lipids have not been fully elucidated and deserve further close study.

Lipid and phospholipid oxidation products (reactive carbonyl species) participate in signaling pathways by modifying proteins. Reactive carbonyls activate the proinflammatory signaling pathway through NF-κB [85] and antioxidant signaling pathway through NRF2 [86]. This may explain the different effects of cardiolipin-coated nanoceria on oxidative metabolism genes.

An interesting result obtained in this study was the activating effect of cardiolipin-coated nanoceria on autophagy. We hypothesize that autophagy is activated by cardiolipin peroxidation products. Autophagy is responsible for removing the effects of oxidative stress, so its activation occurs during increased production of reactive oxygen species and lipid peroxidation [87]. Indeed, products of lipid peroxidation such as 4-HNE (4-hydroxynonenal) and acrolein activate autophagy in rat aortic smooth-muscle cells [88]. Hemin-dependent lipid peroxidation leads to activation of autophagy in endothelial cells [89]. The effect of peroxidation is related to not only to the initial stages of autophagy, where 4-hydroxynonenal and malondialdehyde affect the proteins involved in the initiation of autophagy and the formation of the phagophore, but also to the final stage, degradation, where reactive aldehydes bind to the active center of cathepsins and inactivate their proteolytic functions [87]. However, pronounced peroxidation of phospholipids leads to an overload of peroxidized phospholipids and culminates in the inhibition of autophagy [90]. Regulation of apoptosis and autophagy by reactive oxygen species during lipid peroxidation is very complex and depends on the concentration of ROS and many factors [91].

To summarize, the effects of cardiolipin-coated nanoceria are due to both the nanoceria core and cardiolipin coating. The contribution of cardiolipin is most likely due to cardiolipin peroxidation and the effects of the peroxidation products.

## 5. Conclusions

In human embryonic lung fibroblasts, the effects of cardiolipin-coated nanoceria on oxidative metabolism were shown to be mediated by both nanoceria and cardiolipin. Cell survival, mitochondrial activity, and the proliferative effect were similar for bare and cardiolipin-coated nanoceria. In contrast to bare nanoceria, cardiolipin-coated nanoceria induced intracellular oxidative stress and had short-term activating effects on these genes and DNA damage/repair. The very specific effect of cardiolipin-coated nanoceria is the induction of long-term activation of autophagy. Presumably, the differences in properties of bare and cardiolipin-coated nanoceria are due to lipid peroxidation of cardiolipin. This effect must be taken into account when developing mitochondria-targeted nanoceria pharmaceuticals, since nanoceria may interact with cardiolipin upon contact with the mitochondrial membrane.

## Figures and Tables

**Table 1:** Physicochemical characteristics of CeO_2_ sols.

Sample	Particle Size, nm (Powder X-Ray Diffraction)	Particle Size, nm (Dynamic Light Scattering)	ζ, mV
Bare CeO_2_	2.9 ± 0.3	12.0 ± 0.9	39.6 ± 0.3
CL-coated CeO_2_	5.9 ± 0.4	38 ± 5	−36.6 ± 0.4

**Table 2:** The effects of cardiolipin-coated nanoceria (1.5 µM) on human embryonic lung fibroblasts.

Parameter	1–3 h (Short-Term Effects)	24 h (Middle-Term Effects)	72 h (Long-Term Effects)
Intracellular reactive oxygen species	↑	0	—
NOX4 protein	↑	0	0
NRF2 protein	↑	0	0
NF-κB protein	↓↑	↑	↓
Oxidative DNA modification (8-oxo-dG)	↑	0	↓
DNA double-strand breaks (phos-γH2AX protein)	↑	0	↓
DNA repair (BRCA1 protein)	↓↑	↓	0
Autophagy (LC3 protein)	↓	0	↑
Proliferation (PCNA protein)	↑	0	↑

‘0′ means the absence of a significant difference between the treatment and control values, ‘↑′ means a significant increase in the parameter, ‘↓′ means a significant decrease in the parameter.

## Data Availability

The original contributions presented in the study are included in the article/Supplementary Materials. Further inquiries can be directed to the corresponding author.

## Supplementary Materials

The following supporting information can be downloaded at: https://www.mdpi.com/article/10.3390/biom15010053/s1, Estimation of the ligand:cardiolipin molar ratio; Figure S1: Structure of cardiolipin form bovine heart, polyunsaturated fatty acid residues comprise primarily linoleic acid (the picture is taken from the official website https://www.sigmaaldrich.com/ accessed on 30 December 2024); Figure S2: (a) Transmitted electron images and electron diffraction data (inset) of bare CeO_2_; (b) transmitted electron images and electron diffraction data (inset) of cardiolipin-coated CeO_2_; (c) diffractograms of the dried sols of bare and cardiolipin-coated CeO_2_; Figure S3: Fourier-transform infrared spectra with attenuated total reflection of bare CeO_2_, cardiolipin, and cardiolipin-coated CeO_2_ nanoparticles; Figure S4: UV-vis absorption spectra of cerium dioxide sols; Figure S5: Distributions of hydrodynamic diameters in aqueous colloidal solutions of (a) bare cerium dioxide and (b) cardiolipin-coated cerium dioxide; and Figure S6: Fluorescence spectra (λ_ex_ = 250 nm) of bare and cardiolipin-coated CeO_2_ nanoparticles. Reference [92] is cited in the supplementary materials.
