# Supplementary material for: Lipid Coating Modulates Effects of Nanoceria on Oxidative Metabolism in Human Embryonic Lung Fibroblasts: A Case of Cardiolipin
Source: Biomolecules. 2025 Jan 2;15(1):53. doi: 10.3390/biom15010053 (PMC11764243; doi:10.3390/biom15010053)
Supplement: Supplementary file 1 [file biomolecules-15-00053-s001.zip › biomolecules-3248329-supplementary.pdf]

biomolecules

Supplementary

# Lipid coating modulates effects of nanoceria on oxidative metabolism in human embryonic lung fibroblasts: a case of cardiolipin

**Elena V. Proskurnina, Madina M. Sozarukova, Elizaveta S. Ershova, Ekaterina A. Savinova, Larisa V. Kameneva, Natalia N. Veiko, Maria A. Teplonogova, Vladimir P. Saprykin, Vladimir K. Ivanov, and Svetlana V. Kostyuk**

*Estimation of ligand:cardiolipin molar ratio*

The ligand:$CeO_2$ molar ratio required to ensure the complete coating of the $CeO_2$ nanoparticles was estimated from the particle geometry, assuming its rigid sphere model. This estimate is approximate. The molar ratio was calculated using the formula [1]:

$$ligand: CeO_2 = \frac{3 \cdot a^3}{TPSA \cdot D_{XRD}} = \frac{3 \cdot 5.410^3}{243 \cdot 29} = 0.07: 1,$$

where $a$ – unit cell parameter of $CeO_2$ (Å), $D_{XRD}$ – diameter of $CeO_2$ nanoparticles (Å), according to X-ray diffrctometry data, TPSA – topological polar surface area of ligand (Å$^2$) [https://pubchem.ncbi.nlm.nih.gov/compound/Cardiolipin#section=Computed-Properties].

The results indicate that at the molar ratio 1:1 ligand:$CeO_2$ is presented in this study, the surface of the $CeO_2$ nanoparticles is completely covered with the ligand.

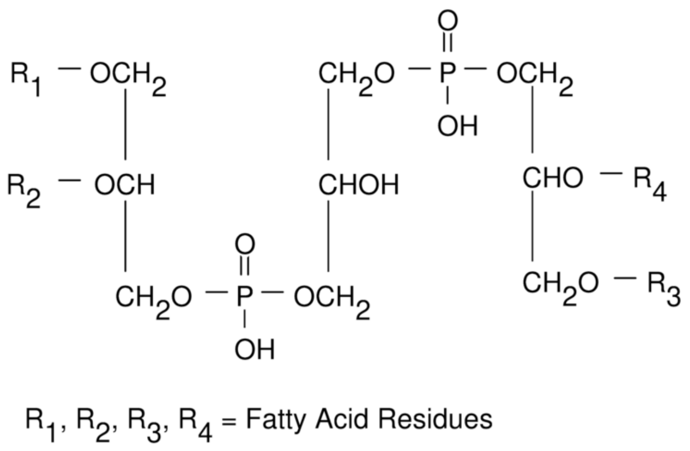


**Figure S1.** Structure of cardiolipin form bovine heart, polyunsaturated fatty acid residues comprise primarily linoleic acid; the picture is taken from the official website https://www.sigmaaldrich.com/.

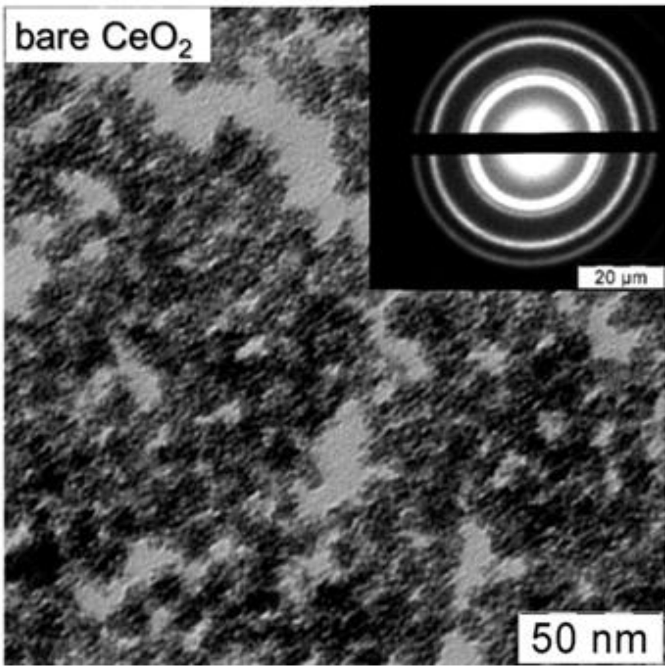


(**a**)

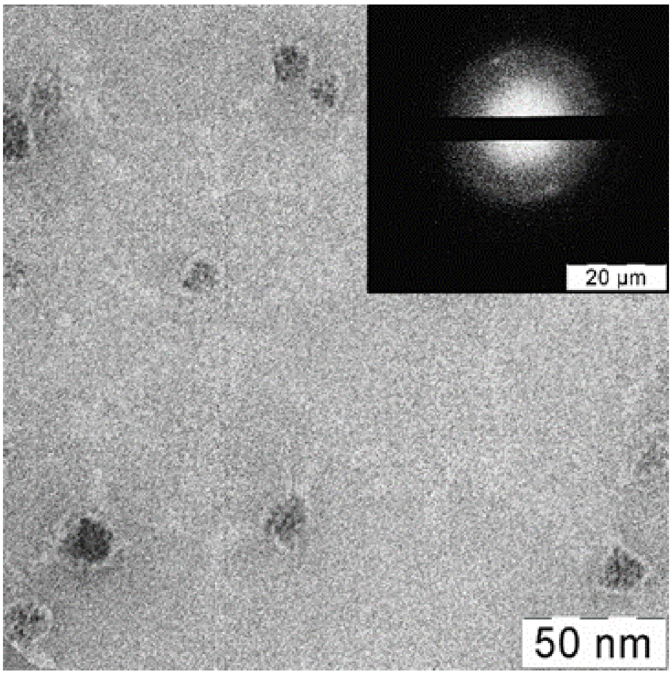


(**b**)

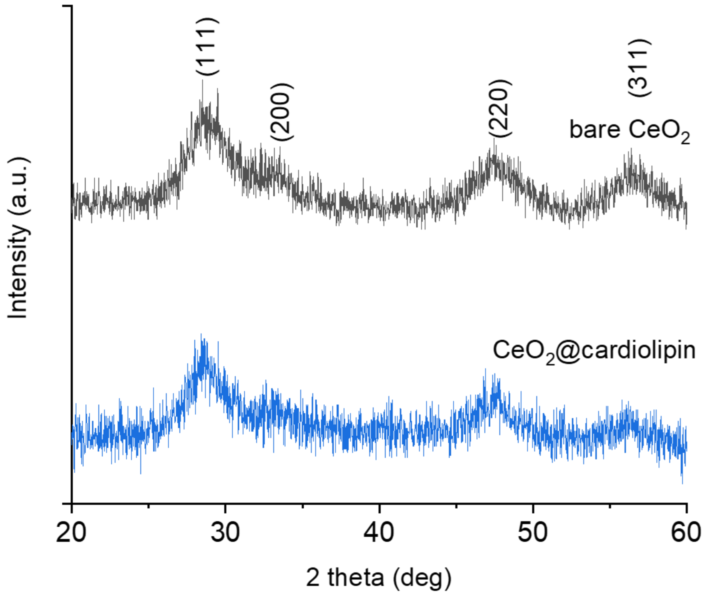


(**c**)

**Figure S2.** (**a**) Transmitted electron images and electron diffraction data (inset) of bare $CeO_2$; (**b**) transmitted electron images and electron diffraction data (inset) of cardiolipin-coated $CeO_2$; (**c**) diffractograms of the dried sols of bare and cardiolipin-coated $CeO_2$.

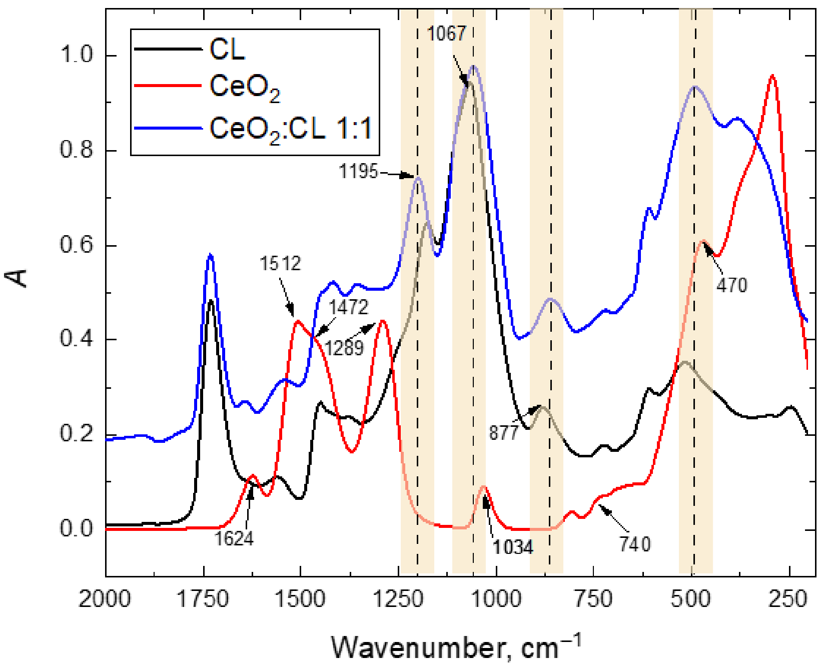


**Figure S3.** Fourier-transform infrared spectra with attenuated total reflection of bare $CeO_2$, cardiolipin, and cardiolipin-coated $CeO_2$ nanoparticles.

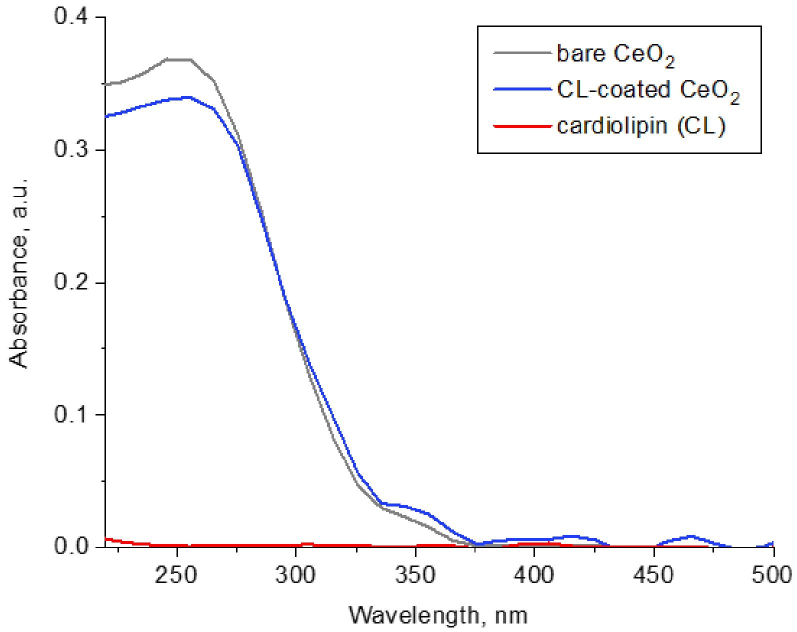


**Figure S4.** UV-vis absorption spectra of cerium dioxide sols.

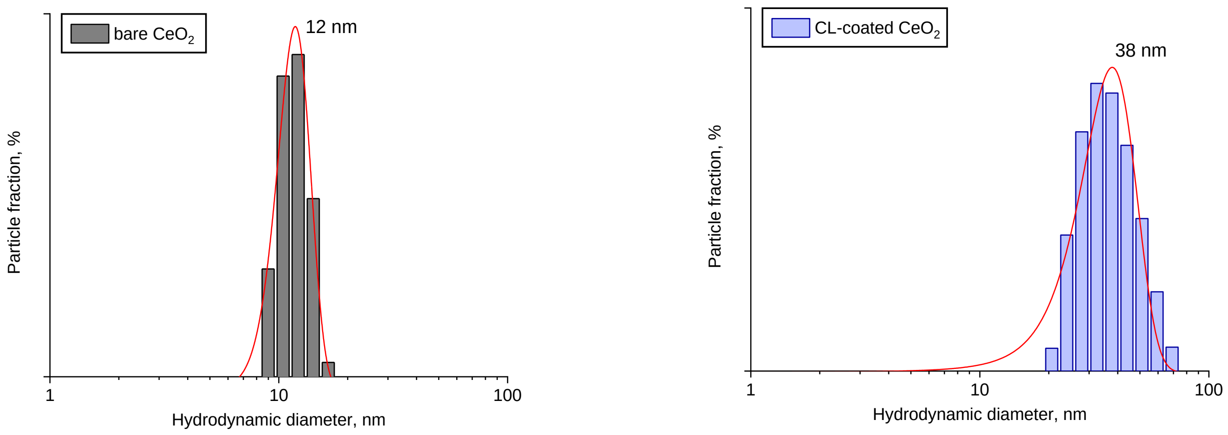


(**a**) (**b**)

**Figure S5.** Distributions of hydrodynamic diameters in aqueous colloidal solutions of (**a**) bare cerium dioxide and (**b**) cardiolipin-coated cerium dioxide.

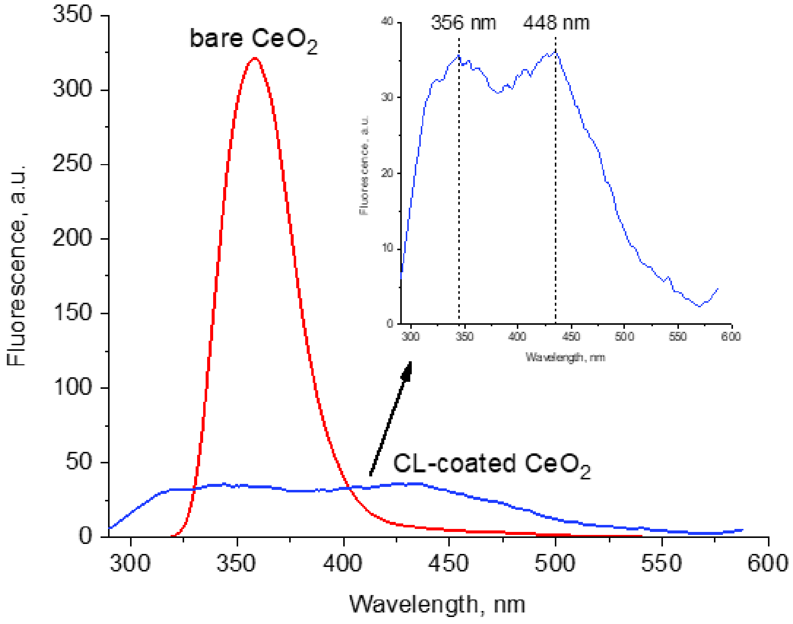


**Figure S6.** Fluorescence spectra ($\lambda_{ex}$ = 250 nm) of bare and cardiolipin-coated $CeO_2$ nanoparticles.

## References

1. Filippova, A.D.; Baranchikov, A.E.; Teplonogova, M.A.; Savintseva, I.V.; Popov, A.L.; Ivanov, V.K. Ligand-to-Metal Ratio Governs Radical-Scavenging Ability of Malate-Stabilised Ceria Nanoparticles. *Nanomaterials* **2024**, *14*, 1908, doi:10.3390/nano14231908.
